# Chondrosarcome né sur un ostéochondrome solitaire: à propos d’un cas

**DOI:** 10.11604/pamj.2019.32.143.15533

**Published:** 2019-03-26

**Authors:** Hafsa Chahdi, Amal Damiri, Mohamed Reda El Ochi, Mohamed Allaoui, Abderrahmane Al Bouzidi, Mohamed Oukabli

**Affiliations:** 1 Department of Pathology, Military General Hospital Mohammed V, Mohammed V, Souissi University, Hay Riad, Rabat, Morocco

**Keywords:** Osteochondroma, sacroiliac, malignant transformation, chondrosarcoma, Ostéochondrome, sacro-iliaque, transformation maligne, chondrosarcome

## Abstract

Le chondrosarcome est une tumeur osseuse maligne rare. Elle peut être primitive ou secondaire à une transformation maligne d'une tumeur cartilagineuse bénigne sous-jacente. Le chondrosarcome secondaire découlant d'un ostéochondrome solitaire bénin est extrêmement rare et les données montrent que l'incidence rapportée de l'ostéochondrome du bassin est très faible. Nous rapportons le cas d'un patient âgé de 20 ans présentant un chondrosarcome secondaire à la transformation maligne d'un ostéochondrome de l'aile sacro-iliaque droite.

## Abstract (en)

Chondrosarcoma is a rare malignant bone tumor. It can arise de novo or secondary to a malignant transformation of a benign underlying cartilage tumor. Secondary chondrosarcoma arising from solitary benign osteochondroma is extremely rare and data show that the reported incidence of osteochondroma of the pelvis is very low. We here report the case of a 20-year old patient with chondrosarcoma secondary to malignant transformation of an osteochondroma of the right wing of ilium, adjacent to the sacroiliac joint.

## Introduction

Le chondrosarcome est une tumeur à histogénèse cartilagineuse souvent primitive mais dans 10% des cas, elle peut survenir suite à une dégénérescence maligne de tumeurs bénignes préexistantes, principalement l'exostose ostéogénique et le chondrome [1-3]. Nous rapportons le cas d'un chondrosarcome secondaire de l'aile sacro-iliaque droite chez un jeune patient de 20 ans, survenu suite à la dégénérescence d'un ostéochondrome solitaire évoluant depuis 9 mois. Compte tenu de l'augmentation du volume de l'exostose et son caractère douloureux, la transformation maligne a été immédiatement suspectée suite à une extension osseuse et extra-osseuse. Une biopsie chirurgicale a conclut le diagnostic d'un chondrosarcome de bas grade.

## Patient et observation

**Figure 1:**
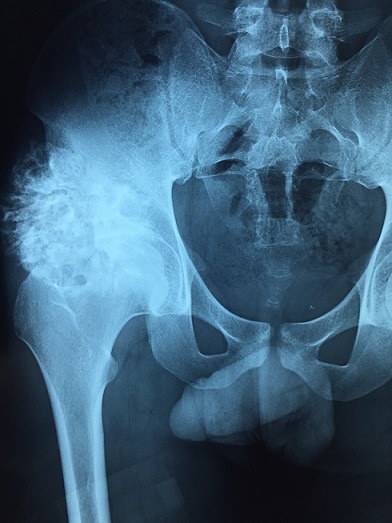
Radiographie antéro-postérieure de la hanche droite montrant une masse en chou-fleur lobulée et calcifiée

**Figure 2:**
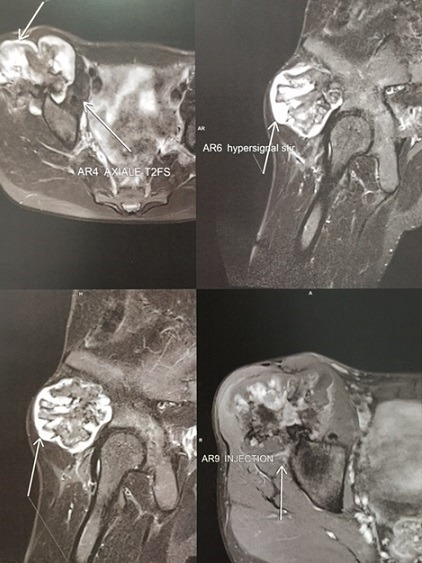
IRM: masse cartilagineuse hétérogène rehaussée après injection faisant suspecter une dégénérescence maligne

**Figure 3:**
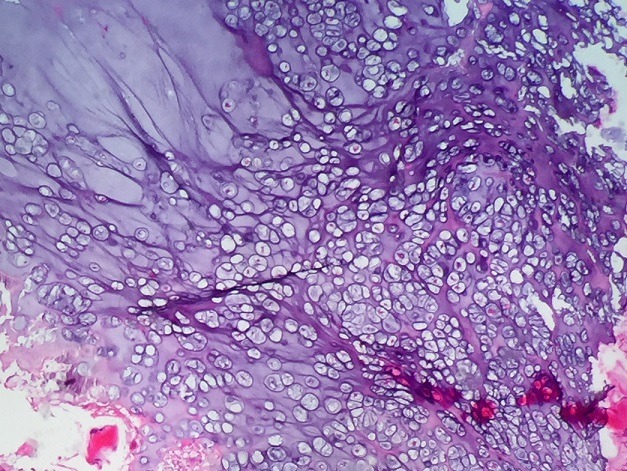
Aspect microscopique du chondrosarcome de bas grade: hémateine-éosine Gx10

**Figure 4:**
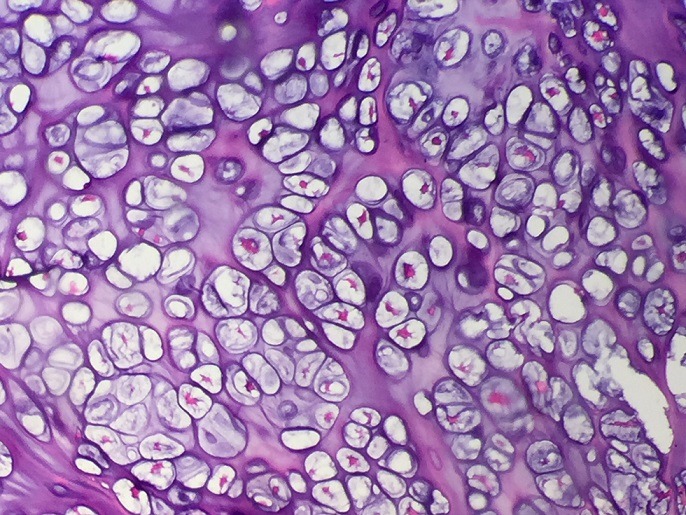
Aspect microscopique mettant en évidence un champ hypercellulaire de chondrocytes désorganisés menus d’atypies nucléaires modérés

Il s'agissait d'un patient âgé de 20 ans sans antécédents pathologiques connus, suivi depuis 9 mois pour une exostose ostéogénique de l'aile iliaque droite. Ayant presenté des douleurs brutales du bassin du coté droit, avec des signes inflammatoires à aggravation progressive sans notion de fièvre ni d'altération de l'état général. À l'examen, on notait une douleur provoquée dans la pression et dans la manœuvre de flexion extension du bassin. Le rachis était flexible. Le reste de l'examen somatique était sans particularités. En biologie, la vitesse de sédimentation était à 80 mm et la CRP est à 18 mg/l. L'hémogramme était normal. La radiographie antéro-postérieure de la hanche droite a révélé une masse en choux-fleur lobulée et calcifiée faisant saillie de l'os iliaque latéral droit (Figure 1). L'imagerie par résonance magnétique du bassin a conclu l'existence d'un ostéochondrome de l'aile iliaque droite avec composante cartilagineuse hétérogène rehaussée après injection faisant suspecter une dégénérescence maligne (Figure 2) sans signes d'envahissement des organes pelviens. Une biopsie chirurgicale a été effectuée et a montré que le tissu avait une apparence grossièrement cartilagineuse. L'examen microscopique a mis en évidence une prolifération dense hypercellulaire de chondrocytes désorganisés menues d'atypies nucléaires modérées et de quelques figures mitotiques, certains chondrocytes étaient augmentés de taille, binucléés et dodus (Figure 3, Figure 4). Le diagnostic de chondrosarcome de bas grade a été posé. Le patient était donc candidat pour une large résection. Sur la base de ce diagnostic, aucun traitement adjuvant n'a été administré. Le patient a depuis été surveillé avec une surveillance pulmonaire de routine par un scanner et une surveillance locale avec des radiographies et un examen physique. Il est maintenant à un an de la résection chirurgicale et se porte bien sans aucune limitation fonctionnelle évidente ou signalée.

## Discussion

L'ostéochondrome solitaire (exostose ostéocartilagineuse ou exostose) représente 20 à 50% des tumeurs bénignes de l'os et 10 à 15% de l'ensemble des tumeurs osseuses [1, 2, 4]. L'âge de diagnostic se situe entre 10 et 15 ans avec une prédilection masculine [2]. La localisation de la maladie exostosante rend compte de sa pathogénie. Cette localisation se fait essentiellement sur les os à croissance enchondrale préférentiellement au niveau des extrémités des os long [2, 3]. Les exostoses du squelette axial (rachis, cote) sont rarement rapportées [4]. La découverte de ces lésions asymptomatiques est dans la plus part du temps fortuite. Les formes symptomatiques se révèlent avant l'âge de 20 ans [4-6]. L'augmentation de volume et la douleur sont les symptômes les plus fréquents et traduisent des phénomènes variables (compressions des éléments vasculo-nerveux et ou la dégénérescence) [5]. La radiographie standard permet le diagnostic de certitude dans la majorité des cas, même en l'absence de confirmation anatomopathologiques. D'autres moyens d'imagerie comme l'imagerie par résonance magnétique et la scintigraphie osseuse peuvent apporter une aide précieuse au diagnostic [4]. La dégénérescence maligne est la complication la plus redoutable de l'ostéochondrome. Il s'agit le plus souvent d'un chondrosarcome de bas grade, bien que de rares cas d'autres types de sarcomes soient rapportés [4, 7, 8]. Cette dégénérescence maligne est tardive survenant toujours après l'âge de 20 ans, autour de 40 à 50 ans [1, 3]. Elle est plus fréquente sur le pelvis (42%), l'extrémité supérieure du fémur (18%) et l'épaule (18%) [4]. La manifestation clinique est la reprise évolutive d'une exostose jusque-là stable, qui devient douloureuse et qui augmente de taille [1, 8]. La transformation maligne se fait au sein de la coiffe cartilagineuse [6, 9], dont l'augmentation de l'épaisseur est le signe le plus précoce, et qu'il faut dépister par l'échographie ou par l'imagerie par résonnance magnétique (IRM). D'autres signes radiologiques qui doivent faire suspecter la dégénérescence, tels que la présence de calcifications irrégulières, hétérogènes dépassant les limites de l'exostose, plus nombreuses et plus grosses d'un clichée à l'autre, ou l'existence d'une masse des tissus mous entourant l'exostose. La scintigraphie osseuse peut aider à identifier des ostéochondromes actifs qui sont le siège d'une fixation intense du marqueur radioactif [5]. Cependant, elle ne permet pas de différencier les ostéochondromes bénins siège d'une formation active d'os enchondral, des exostoses dégénérées. En outre, une scintigraphie normale n'exclut pas une dégénérescence maligne [10]. En fait, aucun critère n'est absolu et toute exostose qui fait secondairement parler d'elle et qui est douteuse sur le plan radiologique doit être excisée. C'est l'examen anatomopathologique de l'ensemble de la pièce qui apportera la preuve définitive et sure du diagnostic. Le traitement chirurgical est indiquée à chaque fois que l'exostose est volumineuse, douloureuse et /ou dégénérée et consiste à reséquer complètement la tumeur

## Conclusion

La transformation maligne en un chondrosarcome, même rare, est la complication la plus redoutable pouvant survenir dans les lésions cartilagineuses bénignes. La plupart de ces chondrosarcomes secondaires sont de grade histologique faible et de faible capacité métastatique. Bien qu'elle soit rare dans l'ostéochondrome solitaire du bassin, la transformation maligne doit toujours être prise en considération. Les changements des particularités cliniques et les aspects radiologiques de l'ostéochondrome doivent être considérés avec beaucoup de précautions. Le diagnostic de certitude est basé sur l'examen histopathologique.

## Conflits d’intérêts

Les auteurs ne déclarent aucun conflit d'intérêts.
